# Estimating the phase diagrams of deep eutectic solvents within an extensive chemical space

**DOI:** 10.1038/s42004-024-01116-3

**Published:** 2024-02-12

**Authors:** Adroit T. N. Fajar, Takafumi Hanada, Aditya D. Hartono, Masahiro Goto

**Affiliations:** 1 Department of Applied Chemistry, Graduate School of Engineering, Kyushu University, 744 Motooka, Fukuoka, 819-0395 Japan; 2 Center for Energy Systems Design (CESD), International Institute for Carbon-Neutral Energy Research (WPI-I2CNER), Kyushu University, 744 Motooka, Fukuoka, 819-0395 Japan; 3 Department of Applied Chemistry, Graduate School of Technology, Industrial and Social Science, Tokushima University, 2-1 Minamijosanjima, Tokushima, 770-8506 Japan; 4 Mathematical Modeling Laboratory, Department of Agro-environmental Sciences, Faculty of Agriculture, Kyushu University, 744 Motooka, Fukuoka, 819-0395 Japan

**Keywords:** Ionic liquids, Computational chemistry

## Abstract

Assessing the formation of a deep eutectic solvent (DES) necessitates a solid-liquid equilibrium phase diagram. Yet, many studies focusing on DES applications do not include this diagram because of challenges in measurement, leading to misidentified eutectic points. The present study provides a practical approach for estimating the phase diagram of any binary mixture from the structural information, utilizing machine learning and quantum chemical techniques. The selected machine learning model provides reasonably high accuracy in predicting melting point (*R*^*2*^ = 0.84; *RMSE* = 40.53 K) and fusion enthalpy (*R*^*2*^ = 0.84; *RMSE* = 4.96 kJ mol^−1^) of pure compounds upon evaluation by test data. By pinpointing the eutectic point coordinates within an extensive chemical space, we highlighted the impact of the mole fractions and melting properties on the eutectic temperatures. Molecular dynamics simulations of selected mixtures at the eutectic points emphasized the pivotal role of hydrogen bonds in dictating mixture behavior.

## Abstract (web summary)

Deep eutectic solvents are promising, sustainable alternatives for diverse applications, including separation, gas capture, electrodeposition, or nanomaterial synthesis, but our fundamental understanding of the nature of these solvents remains limited. Here, the authors report a practical approach for estimating the solid–liquid equilibrium phase diagram of any binary mixture from structural information, using machine learning and quantum chemical techniques.

## Introduction

Solvents play a pivotal role in facilitating chemical processes in vital industries, including pharmaceutical production, oil refining, and fine chemical production^1^. In 2020, the global market for common organic solvents was USD 43,845.7 million, with projections estimating it will reach USD 67,837.8 million by 2028^2^. Regrettably, the majority of organic solvents in use today pose environmental and health risks, thus there is a need to develop more sustainable alternatives. Deep eutectic solvents (DESs) have emerged as a potential solution for the environmental and health issues observed with current solvents. First described by Abbott et al.^3,4^, DESs have since captured considerable attention as promising green solvents^5^. Literature reports have underscored the versatility of DESs, highlighting their potential in diverse applications, including separation, gas capture, electrodeposition, batteries, biomass processing, medical research, and nanomaterial synthesis^6^.

While the potential applications of DESs are vast, a fundamental understanding of the nature of DESs remains limited^7^. The very definition of DES is nebulous, leading to divergent interpretations among researchers^8^. Abranches and Coutinho have highlighted several prevalent misconceptions in the literature^9^, including: (i) viewing DESs as analogous to ionic liquids (ILs); (ii) viewing the depression of the melting point as a unique characteristic of DESs; and (iii) presuming DESs form at fixed stoichiometric ratios. These misconceptions risk diverting DES research down incorrect avenues and require urgent redress. We concur with Martins et al.^10^, who defined DESs from the intrinsic thermodynamic traits, i.e., DESs are a subset of eutectic mixtures that exhibit negative deviations from thermodynamic ideality. The concept of a eutectic mixture, i.e., a mixture with a melting point lower than its individual components, is a basic principle in physical chemistry, recognized long before the advent of DESs^11^.

The DES definition proposed by Martins et al. is favored by many researchers^7,8,12–14^ because it clearly distinguishes ideal eutectic and deep eutectic phenomena from a thermodynamic standpoint. Consequently, a solid–liquid equilibrium (SLE) phase diagram is imperative for evaluating DES formation. While an SLE phase diagram is important for DES assessment, the challenges of obtaining this diagram are evident. Acquiring the melting points of a DES across a full spectrum of molar ratios, typically executed using differential scanning calorimetry^15^, is both labor-intensive and susceptible to moisture interference^16^. Additionally, many DESs derived from natural organic compounds can decompose before the melting properties can be ascertained^17^. Although computational techniques rooted in the conductor-like screening model for real solvents (COSMO-RS) offer some solutions^18^, the software mandates that users input melting property data (e.g., melting point and fusion enthalpy) of the pure components. Such data are often not available, particularly when the DES components contain novel chemical structures. Given these challenges, a practical method for estimating SLE phase diagrams is vital to advancing DES research. Employing such a method can also unlock the chemical potential of DESs, of which only a fraction has been harnessed to date.

Machine learning (ML), a subset of artificial intelligence that enables computers to make accurate predictions based on data patterns, holds the potential to address the challenge of estimating phase diagrams. Recent efforts have applied ML to predict the physical properties of DESs, including density^19,20^ and viscosity^21–23^. These ML models were developed using state-of-the-art datasets reported in the literature. However, preparing ML models to predict SLE phase diagrams of DESs is particularly challenging due to the scarcity of training data. Therefore, implementing ML for SLE phase diagram estimation requires innovative approaches, such as combining ML with COSMO-RS and integrating the results in alignment with thermodynamic principles.

In the present study, we present a practical method for estimating the SLE phase diagrams of DESs, leveraging both ML predictions and quantum chemical (QC) calculations. This strategy enabled a systematic exploration of the expansive chemical space of DESs, relying solely on structural information. Focusing on type V DES, we elucidated the SLE phase diagrams for 3000 mixtures and examined the associated ideal eutectic and deep eutectic behaviors. Furthermore, we conducted molecular dynamics (MD) simulations for selected mixtures at the eutectic mole fractions and temperatures to probe the intrinsic interactions within DESs.

## Results and discussion

### The thermodynamic context

When two immiscible or partially immiscible solid compounds mix under isobaric conditions, the melting points will generally decrease, as characterized by the equation^9,10,24,25^:

1 $${{{{{\rm{ln}}}}}}\left({x}_{i}{\gamma }_{i}\right)=\frac{{\Delta }_{{{{{\rm{fus}}}}}}{H}_{i}}{R}\left(\frac{1}{{T}_{m,i}}-\frac{1}{{T}_{i}}\right)$$

In this equation, ${x}_{i}$, ${\gamma }_{i}$, ${\Delta }_{{fus}}{H}_{i}$, ${T}_{m,i}$, and ${T}_{i}$ represent the mole fraction, activity coefficient, fusion enthalpy (J mol^–1^), pure compound melting point (*K*), and melting point in the mixture (*K*) of component $i$, respectively. $R$ is the ideal gas constant, given as 8.3145 J mol^−1^ K^−1^. Calculating for ${T}_{i}$ across a range of values for ${x}_{i}$ from 0 to 1 produces a melting curve for each mixture component. In a binary mixture, the eutectic point emerges where the melting curves of the two constituents intersect, with the *x* axis and *y* axis delineating the associated eutectic mole fraction ${x}_{E}$ and eutectic temperature ${T}_{E}$, respectively (refer to Fig. S1). Conventionally, if intercomponent interactions match the intracomponent interactions, the mixture exhibits ideal behavior, allowing ${\gamma }_{i}$ to be set at 1. However, real eutectic mixtures can display either a positive (${\gamma }_{i}$ > 1) or negative (${\gamma }_{i}$ < 1) deviation from this ideality. The latter characteristic defines a DES, as proposed by Martins et al.^10^. Accordingly, SLE phase diagrams for both ideal and real mixtures can be derived by inputting the parameters ${T}_{m,i}$, ${\Delta }_{{fus}}{H}_{i}$, and ${\gamma }_{i}$ in Eq. (1). It should be noted that Eq. (1) disregards the value of the molar heat capacity, ${\Delta }_{m}{C}_{i}$ because its impact is minor compared with that of the other parameters (see equation S1).

### Prediction of melting properties

One of the main challenges in employing ML techniques for DES research is the perceived scarcity of extensive datasets, especially those relating to SLE phase diagrams. This perception largely arises from an approach that views DESs as a novel class of compounds, rather than as mixtures. When building ML models tailored to DESs, researchers have often depended on training data sourced solely from previous DES-specific studies, which are relatively limited. In contrast, the melting curves in SLE phase diagrams can be charted individually for each component. Thus, the associated melting properties, ${T}_{m,i}$ and ${\Delta }_{{fus}}{H}_{i}$, can be predicted using ML models trained on datasets of pure compounds, which are abundantly available in the literature^26,27^.

**Fig. 1:**
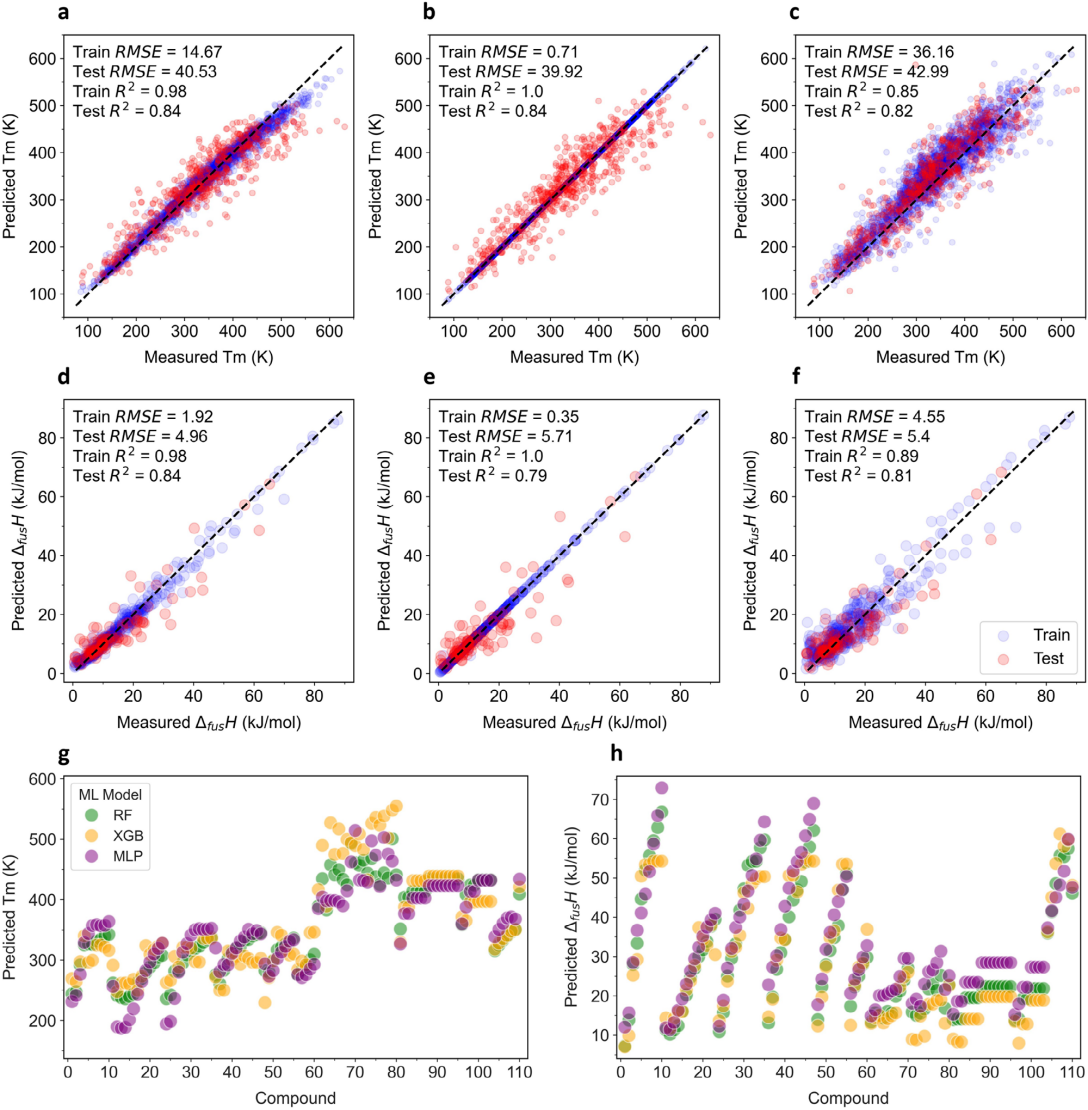
Constructing ML models to predict the melting properties of pure compounds. Parity plots corresponding to training and test data of ML models to predict the ${T}_{m,i}$ value: **a** RF; **b** XGB; and **c** MLP. Parity plots corresponding to training and test data of ML models to predict the ${\triangle }_{{fus}}{H}_{i}$ value: **d** RF; **e** XGB; and **f** MLP. Predicted **g** ${T}_{m,i}$ and **h** ${\triangle }_{{fus}}{H}_{i}$ values for the 110 selected compounds (60 HBAs and 50 HBDs). Source data are provided as a Source Data file.

Figure 1a–c shows parity plots of the training and test data of three ML models developed in this study: random forest (RF); extreme gradient boosting (XGB); and multilayer perceptron (MLP), to predict the ${T}_{m,i}$ parameter. All these models demonstrated a robust learning ability using the prescribed melting point dataset, as evidenced by the low root mean square error (RMSE) and high coefficient of determination (*R*^2^) values. For the training data, XGB recorded the smallest RMSE value (0.71 K), followed by RF (14.67 K) and MLP (36.16 K). A similar trend emerged for the R^2^ values: XGB (1.0) > RF (0.98) > MLP (0.85). When evaluating the test data, all the models consistently gave low RMSE (≈40 K) values and high *R*^2^ (≈0.83) figures, indicating their good predictive ability. Cross-validation analysis further substantiated the reliability of the RF and XGB models, both returning RMSE and *R*^2^ scores of 40 K and 0.82, respectively (Fig. S2a, b). For the MLP model, a sharp decline in the loss function throughout the learning history suggested a swift adaptation to data patterns (Fig. S2c). These superior evaluation scores across the board indicated the aptness of the selected dataset, molecular descriptor, and model design, supporting their validity for use in predicting the melting points of novel or uncharted compounds.

Figure 1e–g shows parity plots of ML models designed to predict the ${\Delta }_{{fus}}{H}_{i}$ parameter. In training data performance, XGB had the best performance with RMSE = 0.35 kJ mol^−1^ and *R*^2^ = 1.0, then RF (RMSE = 1.92 kJ mol^−1^, *R*^2^ = 0.98) and MLP (RMSE = 4.55 kJ mol^−1^, *R*^2^ = 0.89). However, this order shifted when evaluating the test data: RF (RMSE = 4.96 kJ mol^−1^, *R*^2^ = 0.84); followed closely by MLP (RMSE = 5.40 kJ mol^−1^, *R*^2^ = 0.81); and XGB (RMSE = 5.71 kJ mol^−1^, *R*^2^ = 0.79). Notably, the performance of RF and XGB was similar based on cross-validation analysis, while MLP demonstrated a rapid data learning ability (Fig. S2e–g). Given the outstanding results across all the models, there exists the potential to confidently predict the fusion enthalpy of novel or as-yet-unstudied compounds.

Traditionally, mixtures comprising a hydrogen-bond acceptor (HBA) and a hydrogen-bond donor (HBD) have been considered to be likely candidates for forming a DES. Thus, we identified 60 potential HBAs and 50 potential HBDs. The names and simplified molecular-input line-entry system (SMILES) representations of these compounds can be found in Table S1. Our selection of HBAs contained both strong (phosphine oxide, sulfinyl, and urea) and weaker (thiourea) HBA groups, with varied alkyl chain lengths, for a systematic study. The HBDs were largely natural organic compounds, including amino acids, sugars, and fatty acids. Merging these 60 HBAs with the 50 HBDs yielded a total of 3000 possible mixtures. It was anticipated that most of these combinations would result in the formation of type V DES, i.e., DESs obtained from the mixtures of non-ionic compounds^28–30^.

Figure 1g, h shows the predictions for the ${T}_{m,i}$ and ${\Delta }_{{fus}}{H}_{i}$ values using RF, XGB, and MLP models. A discernible trend in the melting point and fusion enthalpy values was evident for the HBAs (compounds 1–60). This trend was aligned with the length of the alkyl chains in each group, specifically, longer alkyl chains corresponded to higher ${T}_{m,i}$ and ${\Delta }_{{fus}}{H}_{i}$ values. This observation was in accordance with basic chemistry principles, indicating the ML models were likely to predict the real values. In contrast, for the HBDs (compounds 61–110), a clear trend was not observed, likely because the chosen HBD structures were not systematically selected. Moreover, the predicted ${T}_{m,i}$ and ${\Delta }_{{fus}}{H}_{i}$ values from each of the RF, XGB, and MLP models largely coincided, as evidenced by the frequent overlaps in the scatterplots. This convergence further bolstered our confidence in the ML predictions. Because of the consistent performance of the model across different datasets and evaluative methods, we opted to use the RF model to derive the ${T}_{m,i}$ and ${\Delta }_{{fus}}{H}_{i}$ values.

### Calculation of activity coefficients

To quantify deviations from thermodynamic ideality, QC methodologies such as density functional theory (DFT) and the COSMO-RS model are valuable tools as these methods have been shown to predict ${\gamma }_{i}$ values with good accuracy^31–35^. The COSMO-RS method, in particular, operates by integrating QC calculations of molecular surfaces with statistical thermodynamics, enabling the assessment of intermolecular interactions within a mixture. This approach allows for the accurate prediction of mixture properties such as solubility and phase behavior, utilizing the potential energy profiles between molecules^36^. In the present study, we used ORCA software for DFT calculations and the OpenCOSMO-RS package for COSMO-RS calculations. The OpenCOSMO-RS, introduced by Gerlach and his team, offers an open-source variant of the COSMO-RS model, with the codebase available in both Python and C++ languages^37^.

**Fig. 2:**
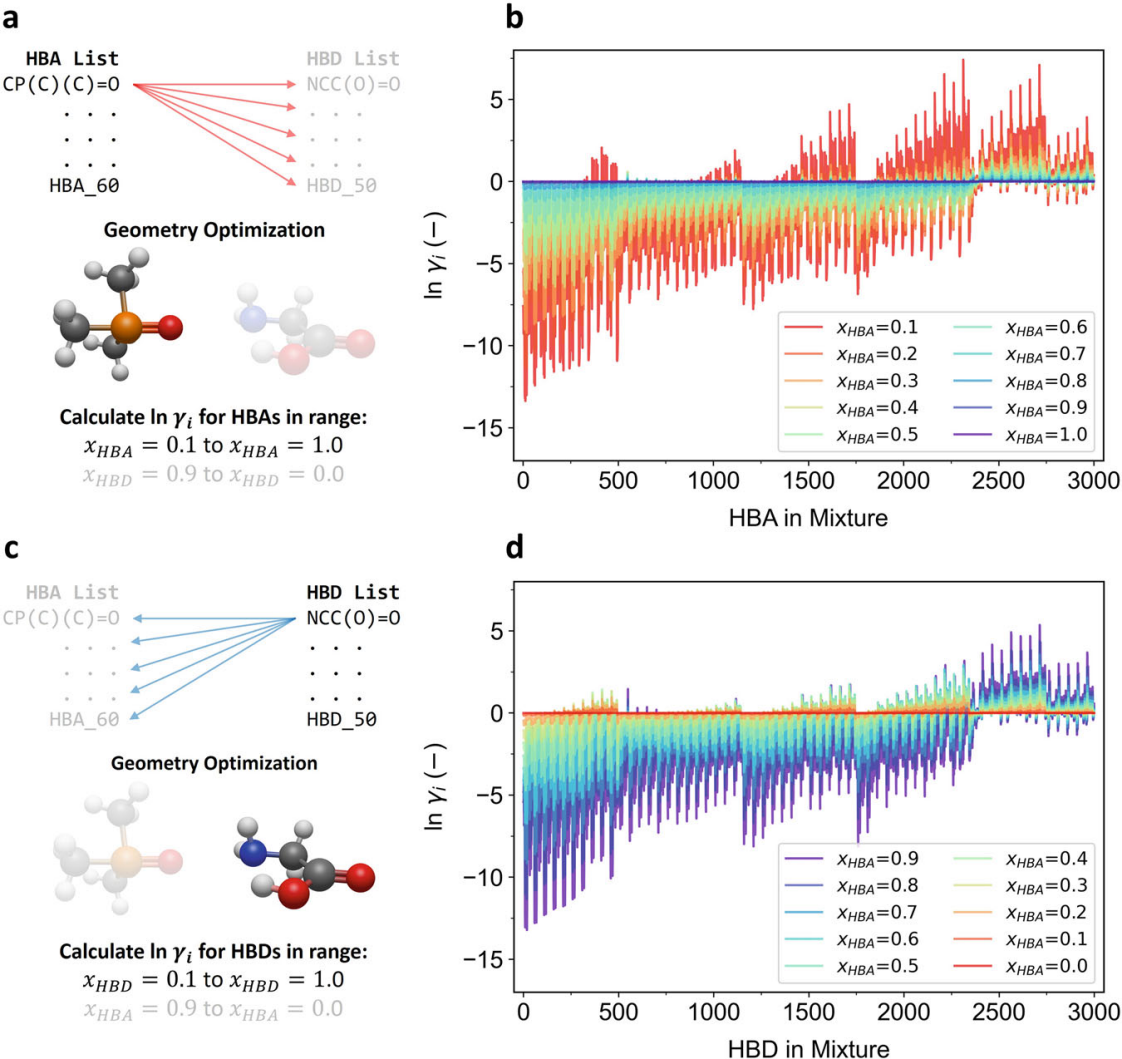
Calculating the activity coefficients of HBAs and HBDs in mixtures. Schematic illustrations of the ${{{{\mathrm{ln}}}}}{\gamma }_{i}$ calculations for **a** HBAs and **c** HBDs. Line plot representations of the calculated ${{{{\mathrm{ln}}}}}{\gamma }_{i}$ values for **b** HBAs and **d** HBDs in the mixtures. Source data are provided as a Source Data file.

Figure 2 shows the calculated ${{{{\mathrm{ln}}}}}{\gamma }_{i}$ values of HBAs and HBDs displayed in panels (b) and (d), respectively, in the mixtures. An illustration of the calculation process is displayed in panels (a) and (c). As anticipated from the nature of real mixtures, the calculated ${{{{\mathrm{ln}}}}}{\gamma }_{i}$ values tended to be closer to 0 as the ${x}_{i}$ values approached 1. Negative$\,{{{{\mathrm{ln}}}}}{\gamma }_{i}$ values indicate a preference for interactions between HBAs and HBDs in a mixture. The calculated ${{{{\mathrm{ln}}}}}{\gamma }_{i}$ values were predominantly negative, suggesting a high likelihood of DES formation from these combinations. However, a sizeable number of the ${{{{\mathrm{ln}}}}}{\gamma }_{i}$ values were positive, indicating that certain mixtures exhibited positive deviations from ideality. It is interesting to note that while the presence of H-bonds was anticipated across all the HBA–HBD combinations, such bonds do not necessarily guarantee negative deviations from ideality. This observation highlights that the correlation between HBA–HBD H-bond formation and the activity coefficients in a mixture is not straightforward. Consequently, relying solely on H-bond metrics as an indicator for DES formation might be misleading. To truly understand the intricacies of DES formation, SLE phase diagrams need to be analyzed across a vast chemical spectrum.

### Estimation of SLE phase diagrams

**Fig. 3:**
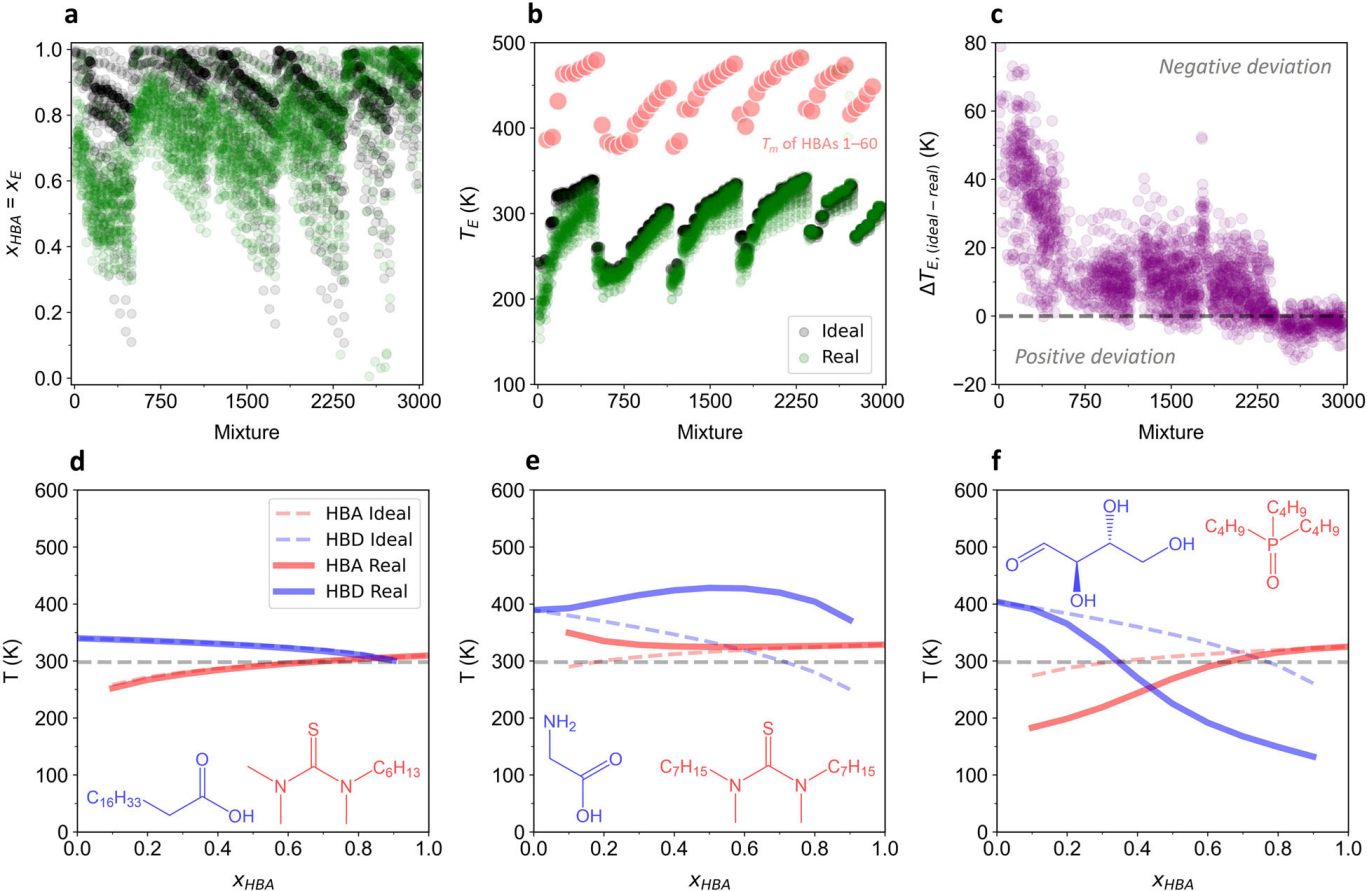
Estimated SLE phase diagrams for the proposed mixtures and analysis of the eutectic points. The distribution of **a** eutectic mole fractions, **b** eutectic temperatures, and **c** eutectic temperature difference between ideal and real models for the investigated mixtures. The red dots in **b** are not part of the graph and are displayed only for visual guidance purposes. SLE phase diagram of **d** Mixture 2996, **e** Mixture 2650, and **f** Mixture 171. The mixture index starts from 0. The inset shows the HBA (red) and HBD (blue) structures. Source data are provided as a Source Data file.

Having determined the parameters ${T}_{m,i}$, ${\Delta }_{{fus}}{H}_{i}$, and ${\gamma }_{i}$ from ML predictions and QC calculations, we estimated SLE phase diagrams for the proposed mixtures. To generate these diagrams, the melting curves of HBAs and HBDs were determined individually by solving for ${T}_{i}$ in Eq. (1) at ${x}_{i}$ = 0.1 to ${x}_{i}$ = 1.0, with increments of 0.1. These curves were then integrated into a single graph, with ${x}_{{HBA}}$ defining the primary *x* axis and ${T}_{i}$ designated as the *y* axis. The curve estimations were carried out for both the ideal mixture model (${\gamma }_{i}$ = 1) and real mixture model (${\gamma }_{i}$ ≠ 1), resulting in the collection of 6000 graphical images and 2 animated videos (Video 1: ideal, Video 2: real) of SLE phase diagrams. The detailed results can be accessed from an external data repository as described in the Methods section. Essential analyses of the resulting SLE phase diagrams are given in Fig. 3.

Figure 3a shows the mole fraction of HBA corresponding to the eutectic mole fraction (${x}_{E}$) for each mixture. A considerable percentage of the ${x}_{E}$ values derived from the ideal mixture model appeared at high HBA fractions (${x}_{{HBA}}$ > 0.8). The real mixture model showed a broader range of ${x}_{E}$ values (${x}_{{HBA}}$ > 0.3), yet most observations were still clustered around high HBA fractions. This pattern underscored the fact that eutectic mixtures typically occur when the molar fractions of the HBAs are comparable to, or surpass, those of the HBDs. This data trend might also explain why numerous reports have claimed successful DES formation at HBD: HBA molar ratios of 1:1, 1:2, and 1:4^6,38,39^. Nevertheless, designing DESs at fixed molar ratios, as is typically the case in IL synthesis, cannot be justified. Such a trend for ${x}_{E}$ values concentrated in a specific mole fraction was not observed in the investigated chemical space. This finding is in line with a previous report debunking “magic compositions” in DESs using ab initio MD simulations^40^. We recommend that DES compositions are consistently described by the molar fractions (${x}_{{HBA}}$ or ${x}_{{HBD}}$), as is common for mixtures, instead of by the molar ratios (${{mol}}_{{HBD}}$: ${{mol}}_{{HBA}}$) to avoid any ambiguities with pure compounds, such as ILs^41^.

Figure 3b shows the distribution of eutectic temperatures (${T}_{E}$) for the investigated mixtures. Interestingly, the data trend appeared to resemble the patterns of the predicted ${T}_{m,i}$ and ${\Delta }_{{fus}}{H}_{i}$ values for the HBAs (Fig. 1g, h, compounds 1–60). This observation suggested that the magnitude of the ${T}_{E}$ value was greatly affected by the melting properties of the HBAs, which was strongly correlated with the alkyl chain lengths. In the real mixture model, numerous ${T}_{E}$ values were found to be lower than those in the ideal mixture model, except for the mixtures 2300–3000. This observation was aligned with the patterns of the ${{{{\mathrm{ln}}}}}{\gamma }_{i}$ values (Fig. 2b, d), indicating a favorable interaction between the HBAs and HBDs in most of the mixtures. Further analyses of the correlations between the ${T}_{m,i}$, ${\Delta }_{{fus}}{H}_{i}$, and ${{{{\mathrm{ln}}}}}{\gamma }_{i}$ values and the ${T}_{E}$ values is shown in Fig. S3, which indicated the HBA melting properties had a considerable effect on the resulting eutectic temperatures.

Figure 3c shows the eutectic temperature difference (${\Delta T}_{E}$) between ideal and real models for the investigated mixtures. The majority of the mixtures within the investigated chemical space had negative deviations from the thermodynamic ideality and thus can be classified as DESs. It should be noted, however, that many eutectic points of the ideal mixture model were located below 298 K (Table 1), i.e., a liquid at room temperature. Therefore, assessing DES formation by merely observing solid-to-liquid transformations would result in the misclassification of ideal eutectic mixtures, or even regular solutions, as DESs, as has been the case in previous studies^13,14^. Basic information from the eutectic point data is summarized in Table 1.

**Table 1:** Basic information from the eutectic point data.

Description	Ideal	Real
(${x}_{E}$, ${T}_{E}$) count	2444	2579
0.1 < ${x}_{E}$ < 0.9	1473	2239
${T}_{E}$ < 298 K	902	1594
${T}_{E}$ max (*K*)	344	485
${T}_{E}$ min (*K*)	232	153

Figure 3d–f shows examples of SLE phase diagrams with melting curves that behave ideally, exhibit positive deviations, and show negative deviations, respectively. Specifically, Fig. 3d displays the SLE phase diagram for Mixture 2996, a combination of 1-hexyl-1,3,3-trimethylthiourea and stearic acid. The melting curves of the real mixture were aligned with those of the ideal model, suggesting that the interactions between 1-hexyl-1,3,3-trimethylthiourea and stearic acid were the same strength as the interactions between the individual compounds. Figure 3e shows the SLE phase diagram for Mixture 2650, comprising 1,3-diheptyl-1,3-dimethylthiourea and glycine. The melting curves showed a positive deviation from ideality, indicating less favorable interactions between the two components than between the individual compounds. Figure 3f shows the SLE phase diagram for Mixture 171, formed by tributylphosphine oxide and erythrose. The melting curves here display a negative deviation, signifying a strong affinity between the two constituents. This particular diagram (Fig. 3f) typifies the SLE phase behavior of a DES. Key details for these mixtures are summarized in Table 2. Additional examples of SLE phase diagrams with varying melting-curve behaviors are shown in Fig. S4 and Table S2. The comparison between estimated SLE phase diagrams for selected cases and their experimental counterparts is presented in Fig. S5, followed by an additional discussion in Note S1. The experimental data were sourced from the recent report by Schaeffer et al.^42^.

**Table 2:** Key information on the investigated mixtures.

Description	Mixture 2996	Mixture 2650	Mixture 171
HBA	1-hexyl-1,3,3-trimethylthiourea	1,3-diheptyl-1,3-dimethylthiourea	tributylphosphine oxide
SMILES	S = C(N(CCCCCC)C)N(C)C	S = C(N(CCCCCCC)C)N(C)CCCCCCC	O = P(CCCC)(CCCC)CCCC
HBD	stearic acid	glycine	erythrose
SMILES	CCCCCCCCCCCCCCCCCC(O) = O	NCC(O) = O	O = C[C@@H]([C@@H](CO)O)O
Behavior	Ideal	Positive	Negative
${x}_{E}$ ideal	0.88	0.60	0.69
${x}_{E}$ real	0.86	0.98	0.44
${T}_{E}$ ideal (*K*)	306	319	315
${T}_{E}$ real (*K*)	306	328	253

### Simulation of DES interactions

MD simulations were carried out for Mixture 2996, Mixture 2650, and Mixture 171 at the respective ${x}_{E}$ and ${T}_{E}$ values. The preliminary stages encompassing energy minimization and system equilibration are shown in Fig. S5. Molecular motions during the simulation can be seen in Video 3 (Mixture 2996), Video 4 (Mixture 2650), and Video 5 (Mixture 171), available on an external data repository as described in the Methods section.

**Fig. 4:**
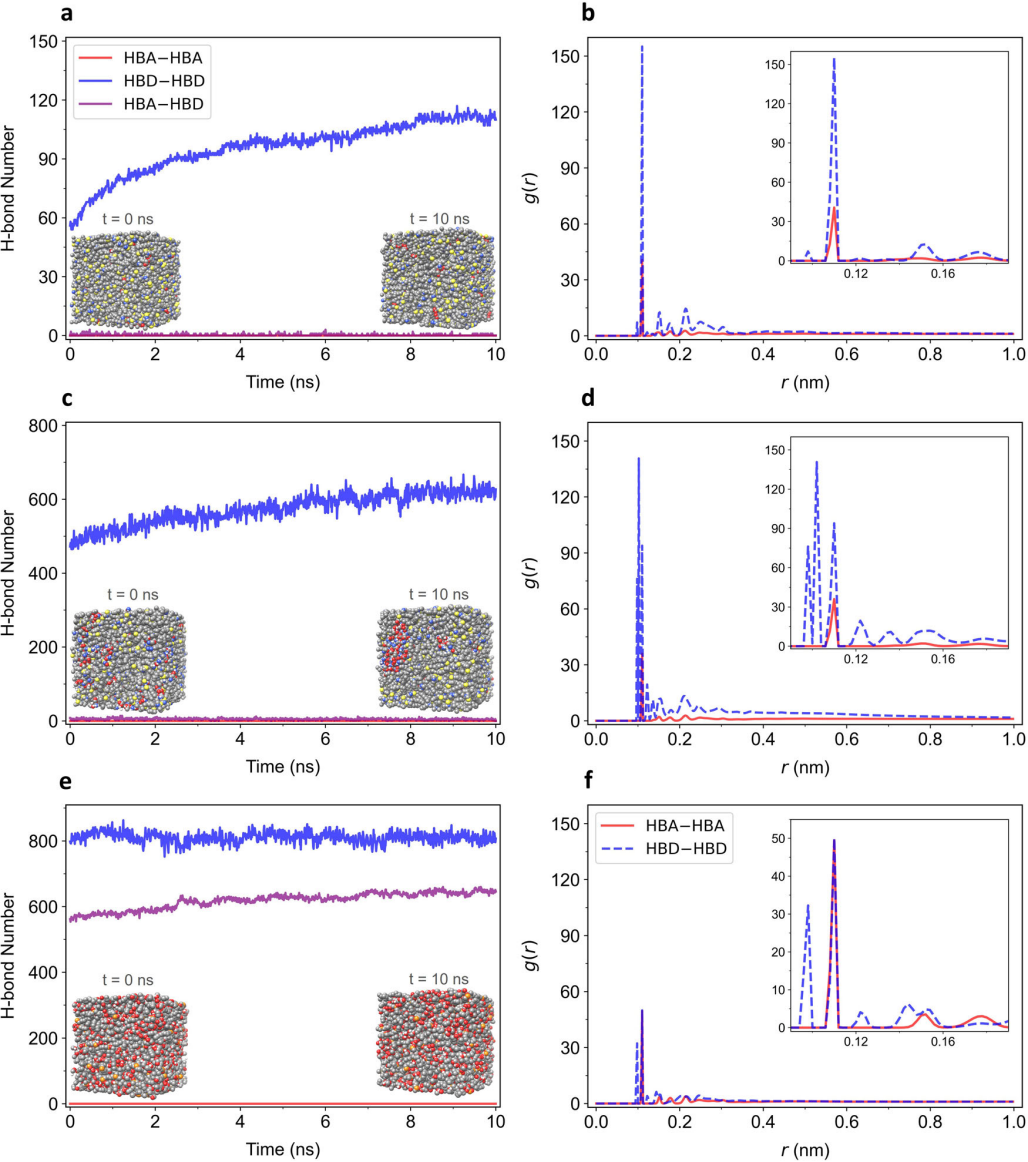
Simulated mixtures to investigate the nature of DES interactions. The number of H-bonds in **a** Mixture 2996, **c** Mixture 2650, and **e** Mixture 171. The simulation box containing a total of 1000 molecules at the initial (*t* = 0 ns) and final time (*t* = 10 ns) is shown in the inset. The mixtures were simulated according to the ${x}_{E}$ and ${T}_{E}$ values. The RDF of **b** Mixture 2996, **d** Mixture 2650, and **f** Mixture 171. The inset shows an enlargement of the values from 0.09–0.19 nm. Source data are provided as a Source Data file.

In the investigated mixtures, H-bonds associated with HBA–HBA interactions were not present because of the absence of hydrogen-donor sites. In Mixture 2996, only ~60 H-bonds associated with HBD–HBD interactions could be observed at the beginning of the simulation, and this number increased to almost 120 over 10 ns (Fig. 4a). This observation suggested that while the interaction between the HBD (stearic acid) molecules in Mixture 2996 was somewhat favorable, it remained fairly modest. The number of H-bonds associated with HBA–HBD interactions was very low (<3), indicating there was a poor interaction between 1-hexyl-1,3,3-trimethylthiourea and stearic acid molecules in the mixture. The radial distribution function (RDF) analysis showed a relatively weak interaction between the HBA molecules and a modest interaction between the HBD molecules, as indicated by the peaks at 0.11 nm (Fig. 4b). In Mixture 2996, even though the strength of the HBA–HBD interactions was weak, the HBA–HBA and HBD–HBD interactions were not particularly strong either. Therefore, the ensemble of these weak interactions allowed Mixture 2996 to behave as an ideal mixture (Fig. 3d).

In Mixture 2650, a considerable amount of H-bonds (~500) associated with HBD–HBD interactions could be observed at the beginning of the simulation (Fig. 4c). Within 10 ns, the number of hydrogen bonds had increased to approximately 600. This increase in H-bonds suggested the formation of HBD regions in the mixture, indicating favorable HBD–HBD interactions and less favorable HBA–HBD interactions. Moreover, there were hardly any H-bonds associated with HBA–HBD interactions. The RDF analysis showed a relatively weak interaction between HBA molecules, as indicated by the small peak at 0.11 nm (Fig. 4d). In contrast, the interactions between the HBD molecules were quite strong, as indicated by the three large peaks at 0.09–0.11 nm. The strength of the HBA–HBD interactions was substantially lower than that of the HBD–HBD interactions, and thus, Mixture 2650 showed a positive deviation from the thermodynamic ideality (Fig. 3e).

Mixture 171 exhibited a markedly high number of H-bonds associated with both HBD–HBD (~800) and HBA–HBD (~600) interactions. The number of H-bonds associated with HBA–HBD interactions appeared to increase over the course of 10 ns, indicating a favorable interaction between the HBA and HBD molecules. The P = O moiety of the tributylphosphine oxide molecule is a good acceptor for H-bonds with the O–H moieties of the erythrose molecule. The RDF analysis showed a few small peaks associated with HBA–HBA and HBD–HBD interactions at 0.09–0.11 nm, indicating that these interactions were not dominant. Although the initial HBD–HBD interactions produced a considerable amount of H-bonds, the ensemble HBA–HBD interactions were favorable and thus led Mixture 171 to undergo a negative deviation from the thermodynamic ideality (Fig. 3f).

## Conclusions

This study presents a practical approach for estimating the SLE phase diagrams of DESs through the integration of ML predictions and QC calculations. ML and QC techniques were used to provide thermodynamic parameters (${T}_{m,i}$, ${\Delta }_{{fus}}{H}_{i}$, and ${\gamma }_{i}$) that dictate the melting behaviors of HBAs and HBDs in mixtures. The ML model demonstrates a fairly high level of precision in predicting the melting point (*R*^2^ = 0.84; *RMSE* = 40.53 K) and fusion enthalpy (*R*^2^ = 0.84; *RMSE* = 4.96 kJ/mol) of pure compounds. Using both ideal and real mixture models, we demonstrated the estimation of SLE phase diagrams for 3000 binary mixtures consisting of systematically selected non-ionic HBAs and HBDs. The analysis of eutectic point coordinates (${x}_{E}$, ${T}_{E}$) over a wide chemical landscape revealed some fundamental insights: (i) Each DES exhibits a distinct eutectic point at a specific composition, diverging from commonly assumed fixed molar ratios; (ii) the magnitude of the ${T}_{E}$ value was strongly correlated with the ${T}_{m,{HBA}}$ and ${\Delta }_{{fus}}{H}_{{HBA}}$ values; (iii) mixtures of HBAs and HBDs frequently formed DESs, but the possibility that the mixtures would behave ideally or deviate positively was not negligible; and (iv) the solid-to-liquid transformation at room temperature or below without further verification of actual deviation from thermodynamic ideality should not be used as the sole identification of a DES. In addition, the MD simulations indicated the importance of the H-bond interactions in a mixture, i.e., fewer H-bonds drives a mixture to behave ideally, predominant H-bonding between HBD molecules leads to positive deviations, and favorable H-bonding between HBA and HBD molecules leads to negative deviations. The developed approach can easily be expanded to a vast chemical space because this method only needs structural information and, therefore, may be used to facilitate the development of DESs and accelerate the discovery of greener solvents for industrial applications. Future research could explore the use of more advanced learning algorithms and larger training datasets to improve prediction accuracy. This should be followed by systematic experimental validation of the estimated SLE phase diagrams. Additionally, the overall estimation process could be simplified by incorporating pretrained ML models into the workflow.

## Methods

### Datasets and molecular descriptors

The melting point dataset, which contained 3041 data points, was sourced from the Bradley Melting Point Dataset, available as open data on Figshare^26^. The fusion enthalpy dataset was manually curated from the CRC Handbook of Chemistry and Physics, 95th Edition, and provided 516 data points on the fusion enthalpy of pure organic compounds^27^. The chemical structures were represented in numerical values using RDKit 2D descriptors, taking SMILES strings as input. These RDKit 2D descriptors encompass 208 features (attributes), which include both physical and structural descriptors^43^.

### ML models

Three ML models using the RF, XGB, and MLP algorithms were constructed and compared. Hyperparameters for each model were fine-tuned either through a grid search or a randomized search, within specific search spaces. For the RF model, the search space encompassed the number of estimators, maximum depth, and maximum features^44^. For the XGB model, this was the number of estimators, maximum depth, and the subsample ratio of columns per tree; and for the MLP model, the number of hidden layers and number of neurons per layer. The models were evaluated using test data and cross-validation analysis. The test data were derived by randomly selecting 20% of the data from the original training data, while cross-validation was executed by splitting the training data into smaller subsets using the k-fold approach. The resulting models were used to predict the melting points and fusion enthalpies of the proposed pure compounds. Construction of these ML models used the scikit-learn, xgboost, and keras libraries^45–47^.

### DFT and COSMO-RS

DFT calculations were executed using the ORCA 5.0 package^48^, following a previously developed workflow^37^. The RDKit package served as the initial tool, generating possible conformers from a given SMILES input^43,49^. The geometries were then optimized using the analytical linearized Poisson–Boltzmann model using the GFN2-xTB calculations^50,51^. The conformers were filtered by an energy window of 6 kcal/mol, clustered by an RMSD window of 1, retaining only those with the lowest energy. Subsequently, COSMO geometry optimizations were performed using the BP86 function with a def2-TZVP(-f) basis set. For the conformer with minimal energy, another COSMO geometry optimization was performed at the BP86 function with a def2-TZVP basis set. This was followed by a single-point calculation at the BP86 function with a def2-TZVPD basis set, producing an “*.orcacosmo*” file. Next, the COSMO-RS model implemented on the OpenCOSMO-RS package was employed to estimate the activity coefficients. The ${{{{\mathrm{ln}}}}}{\gamma }_{i}$ values for HBAs and HBDs in the mixtures were calculated at a temperature of 298.15 K, considering the pure component as the reference state, and using the “*.orcacosmo*” files as input data. For each mixture, the ${{{{\mathrm{ln}}}}}{\gamma }_{i}$ values were calculated at ${x}_{{HBA}}$ = 0.0 to ${x}_{{HBA}}$ = 1.0 with a step size of 0.1. All the calculation processes were controlled by a Python script, enabling a simple and semi-automated workflow. Computation outcomes were archived in the form of a NumPy binary object for subsequent use^45^.

### Coordinates of eutectic points

The coordinates of the eutectic points (${x}_{E}$, ${T}_{E}$) were determined numerically by pinpointing the intersection point of the HBA and HBD melting curves using Brent’s root-finding algorithm^52^. Initially, interpolating functions were generated for each coordinate set, resulting in functions *f1* and *f2*, formulated through cubic spline interpolation. This interpolation enabled value computation at any location on the curve, beyond the scope of the original data points. Subsequently, the function *f3* was delineated as the difference between the functions *f1* and *f2*. In the event of an intersection of the curves, there should exist an *x*-value where *f3* equates to zero, which indicates the intersection point on the original curves. To locate this specific *x*-value, a root-finding operation was undertaken using Brent’s method. The root-finding operation spanned a range determined by the smallest and largest *x* values extracted from both *x1* and *x2*, assuring coverage of the complete range of both curves. Upon convergence of the root-finding operation, an intersection point was determined. The *x*-coordinate of this point aligns with the root of *f3*, and the *y* coordinate was computed using either *f1* or *f2*. In cases where the melting curves lack an intersection, the operation will not converge, and a “None” value was appended to the result. All the calculations were performed by implementing NumPy and SciPy libraries^53,54^, with Matplotlib aiding in the visual representation^55^.

### MD simulations

MD simulations were performed using the GROMACS package with the CHARMM36 all-atom force field^56,57^. The topology and parameters of the molecules were generated by the SwissParam tool^58^. At first, a total of 1000 molecules of the mixtures were placed into a box with a size of 15 × 15 × 15 nm (for Mixture 2996 and Mixture 2650) or 10 × 10 × 10 nm (for Mixture 171). The composition of these mixtures was derived from the ${x}_{E}$ value identified in the associated SLE phase diagram. Subsequently, the system was subjected to energy minimization and equilibration. The equilibration was performed in two steps: (i) under a constant number of particles, volume, and temperature (NVT ensemble) to set the temperature and (ii) under a constant number of particles, pressure, and temperature (NPT ensemble) to set the pressure of the system. The temperature of the simulation system was programmed to follow the ${T}_{E}$ value found in the respective SLE phase diagrams using a Berendsen thermostat^59^. The pressure was restrained at 1.0 bar using a Parrinello-Rahman barostat^60^. Finally, the MD production was carried out for 10 ns with a time step of 2 fs in the respective isothermal and isobar ensembles. The particle mesh Ewald method^61^ with a cutoff distance of 1.0 nm and grid spacing of 0.16 nm was used for the long-range electrostatic interactions. Then, the H-bond number and the RDF were analyzed using the built-in GROMACS functions. Visualizations were facilitated using the UCSF Chimera software package^62^.

### Supplementary information

Supplementary Materials

## Data Availability

All data needed to evaluate the conclusions in this paper are presented in the Manuscript, Supplementary Information, and/or Supplementary Files (Images and Videos). Supplementary Files can be accessed at 10.6084/m9.figshare.23995914. Additional data related to this paper may be requested from the authors.

## Code availability

All codes used in this study were deposited at https://github.com/adroitfajar/eutectic-diagrams.
