# Supplementary material for: Estimating the phase diagrams of deep eutectic solvents within an extensive chemical space
Source: Commun Chem. 2024 Feb 12;7:27. doi: 10.1038/s42004-024-01116-3 (PMC10861527; doi:10.1038/s42004-024-01116-3)
Supplement: Supplementary file 1 — Supplementary Materials [file 42004_2024_1116_MOESM1_ESM.pdf]

SUPPLEMENTARY INFORMATION

Estimating the phase diagrams of deep eutectic solvents within an extensive chemical space

Adroit T.N. Fajar[1,2], Takafumi Hanada[3], Aditya D. Hartono[4], Masahiro Goto[1,*]

[1]*Department of Applied Chemistry, Graduate School of Engineering, Kyushu University, 744 Motooka, Fukuoka 819-0395, Japan.*

[2]*Center for Energy Systems Design (CESD), International Institute for Carbon-Neutral Energy Research (WPI-I2CNER), Kyushu University, 744 Motooka, Fukuoka 819-0395, Japan.*

[3]*Department of Applied Chemistry, Graduate School of Technology, Industrial and Social Science, Tokushima University, 2-1 Minamijosanjima, Tokushima 770-8506, Japan.*

[4]*Mathematical Modeling Laboratory, Department of Agro-environmental Sciences, Faculty of Agriculture, Kyushu University, 744 Motooka, Fukuoka 819-0395, Japan.*

*Corresponding author. Email address: m-goto@mail.cstm.kyushu-u.ac.jp

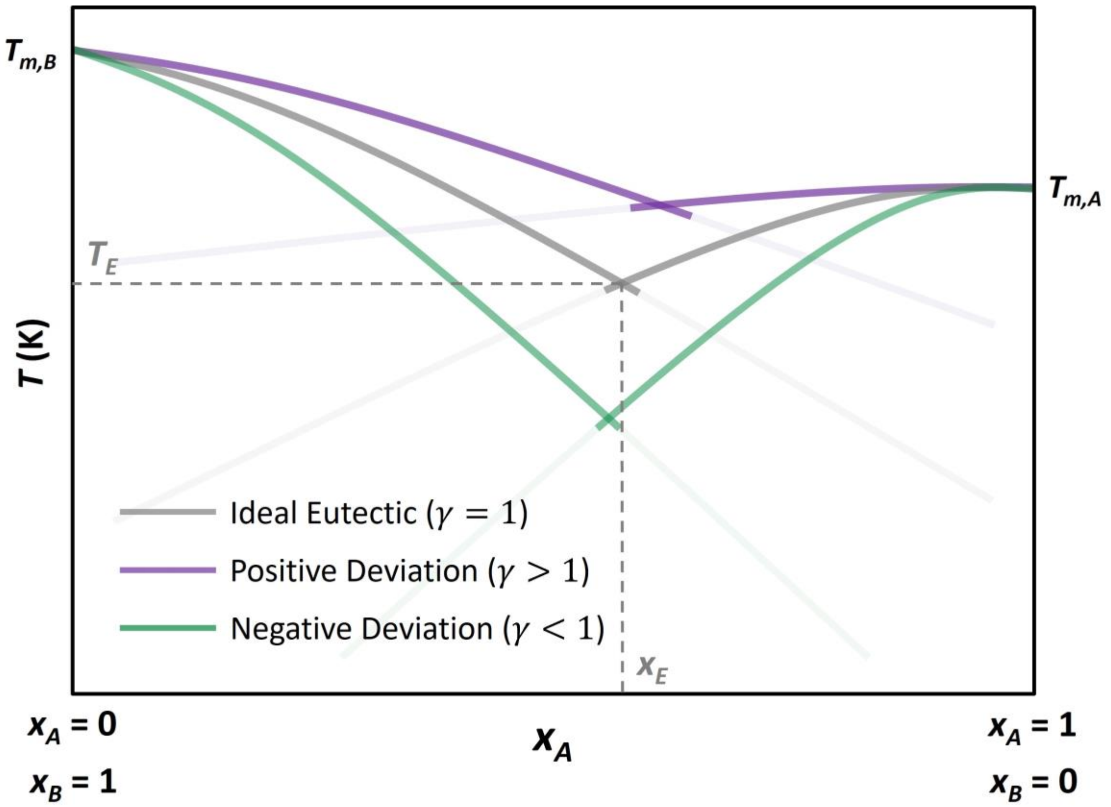

Figure S1. A schematic SLE phase diagram for a binary mixture consisting of compound A and compound B.

Equation S1

If the molar heat capacity parameter was taken into account, equation (1) would expand as follows:

$$ln(x_i\gamma_i) = \frac{\Delta_{fus}H_i}{R}\left(\frac{1}{T_{m,i}} - \frac{1}{T_i}\right) + \frac{\Delta_m C_i}{R}\left(\frac{T_{m,i}}{T_i} - ln\frac{T_{m,i}}{T_i} - 1\right)$$

However, the contribution of molar heat capacity $\Delta_m C_i$ (J mol^{-1} K^{-1}) is negligible compared to that of the fusion enthalpy parameter. Thus, the present study uses equation (1) to determine the melting curves in SLE phase diagrams.

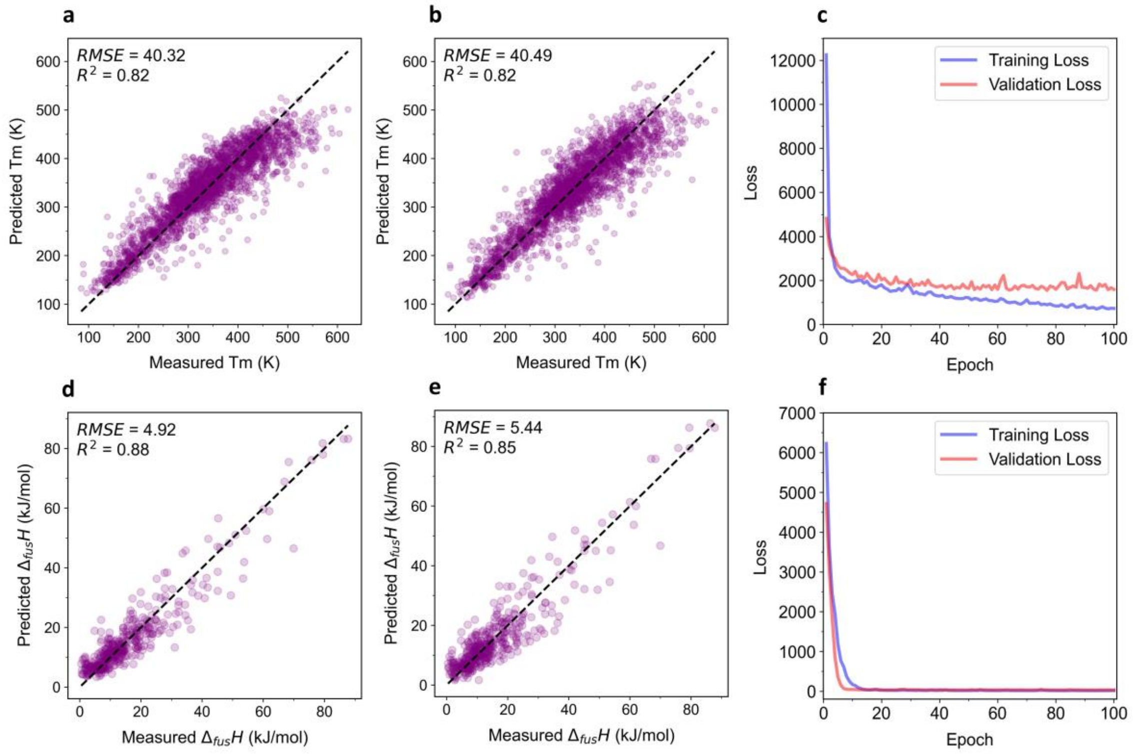

Figure S2. Parity plots corresponding to cross-validation analysis of ML models to predict $T_{m,i}$ value: (**a**) RF and (**b**) XGB. (**c**) The learning history of the MLP model to predict $T_{m,i}$ value. Parity plots corresponding to cross-validation analysis of ML models to predict $\Delta_{fus}H_i$ value: (**d**) RF and (**e**) XGB. (**f**) The learning history of the MLP model to predict $\Delta_{fus}H_i$ value.

Table S1. Names and SMILES representations of the proposed compounds.

HBA	SMILES
trimethylphosphine oxide	CP(C)(C)=O
triethylphosphine oxide	O=P(CC)(CC)CC
tripropylphosphine oxide	O=P(CCC)(CCC)CCC
tributylphosphine oxide	O=P(CCCC)(CCCC)CCCC
tripentylphosphine oxide	O=P(CCCCC)(CCCCC)CCCCC
trihexylphosphine oxide	O=P(CCCCCC)(CCCCCC)CCCCCC
triheptylphosphine oxide	O=P(CCCCCCC)(CCCCCCC)CCCCCCC
trioctylphosphine oxide	O=P(CCCCCCCC)(CCCCCCCC)CCCCCCCC
trinonylphosphine oxide	O=P(CCCCCCCCC)(CCCCCCCCC)CCCCCCCCC
tris(decyl)phosphine oxide	O=P(CCCCCCCCCC)(CCCCCCCCCC)CCCCCCCCCC
(methylsulfinyl)methane	CS(C)=O
(methylsulfinyl)ethane	CS(CC)=O
1-(methylsulfinyl)propane	CS(CCC)=O
1-(methylsulfinyl)butane	CS(CCCC)=O
1-(methylsulfinyl)pentane	CS(CCCCC)=O
1-(methylsulfinyl)hexane	CS(CCCCCC)=O
1-(methylsulfinyl)heptane	CS(CCCCCCC)=O
1-(methylsulfinyl)octane	CS(CCCCCCCC)=O
1-(methylsulfinyl)nonane	CS(CCCCCCCCC)=O
1-(methylsulfinyl)decane	CS(CCCCCCCCCC)=O
1-(methylsulfinyl)undecane	CS(CCCCCCCCCCC)=O
1-(methylsulfinyl)dodecane	CS(CCCCCCCCCCCC)=O
1-(methylsulfinyl)tridecane	CS(CCCCCCCCCCCCC)=O
(ethylsulfinyl)ethane	O=S(CC)CC
1-(propylsulfinyl)propane	O=S(CCC)CCC
1-(butylsulfinyl)butane	O=S(CCCC)CCCC
1-(pentylsulfinyl)pentane	O=S(CCCCC)CCCCC
1-(hexylsulfinyl)hexane	O=S(CCCCCC)CCCCCC
1-(heptylsulfinyl)heptane	O=S(CCCCCCC)CCCCCCC
1-(octylsulfinyl)octane	O=S(CCCCCCCC)CCCCCCCC
1-(nonylsulfinyl)nonane	O=S(CCCCCCCCC)CCCCCCCCC
1-(decylsulfinyl)decane	O=S(CCCCCCCCCC)CCCCCCCCCC
1-(undecylsulfinyl)undecane	O=S(CCCCCCCCCCC)CCCCCCCCCCC
1-(dodecylsulfinyl)dodecane	O=S(CCCCCCCCCCCC)CCCCCCCCCCCC
1-(tridecylsulfinyl)tridecane	O=S(CCCCCCCCCCCCC)CCCCCCCCCCCCC
1,1,3,3-tetramethylurea	O=C(N(C)C)N(C)C
1,3-diethyl-1,3-dimethylurea	O=C(N(C)CC)N(C)CC
1,1,3,3-tetraethylurea	O=C(N(CC)CC)N(CC)CC
1,3-diethyl-1,3-dipropylurea	O=C(N(CC)CCC)N(CC)CCC
1,1,3,3-tetrapropylurea	O=C(N(CCC)CCC)N(CCC)CCC
1,3-dibutyl-1,3-dipropylurea	O=C(N(CCC)CCCC)N(CCC)CCCC
1,1,3,3-tetrabutylurea	O=C(N(CCCC)CCCC)N(CCCC)CCCC
1,3-dibutyl-1,3-dipentylurea	O=C(N(CCCC)CCCCC)N(CCCC)CCCCC

1,1,3,3-tetrapentylurea	O=C(N(CCCCC)CCCCC)N(CCCCC)CCCCC
1,3-dihexyl-1,3-dipentylurea	O=C(N(CCCCC)CCCCCC)N(CCCCC)CCCCCC
1,1,3,3-tetrahexylurea	O=C(N(CCCCCC)CCCCCC)N(CCCCCC)CCCCCC
1,3-diheptyl-1,3-dihexylurea	O=C(N(CCCCCC)CCCCCCC)N(CCCCCC)CCCCCCC
1,1,3,3-tetramethylthiourea	S=C(N(C)C)N(C)C
1,3-diethyl-1,3-dimethylthiourea	S=C(N(CC)C)N(C)CC
1,3-dimethyl-1,3-dipropylthiourea	S=C(N(CCC)C)N(C)CCC
1,3-dibutyl-1,3-dimethylthiourea	S=C(N(CCCC)C)N(C)CCCC
1,3-dimethyl-1,3-dipentylthiourea	S=C(N(CCCCC)C)N(C)CCCCC
1,3-dihexyl-1,3-dimethylthiourea	S=C(N(CCCCCC)C)N(C)CCCCCC
1,3-diheptyl-1,3-dimethylthiourea	S=C(N(CCCCCCC)C)N(C)CCCCCCC
1,3-dimethyl-1,3-dioctylthiourea	S=C(N(CCCCCCCC)C)N(C)CCCCCCCC
1-ethyl-1,3,3-trimethylthiourea	S=C(N(CC)C)N(C)C
1,1,3-trimethyl-3-propylthiourea	S=C(N(CCC)C)N(C)C
1-butyl-1,3,3-trimethylthiourea	S=C(N(CCCC)C)N(C)C
1,1,3-trimethyl-3-pentylthiourea	S=C(N(CCCCC)C)N(C)C
1-hexyl-1,3,3-trimethylthiourea	S=C(N(CCCCCC)C)N(C)C

**HBD**	**SMILES**
Glycine	NCC(O)=O
Alanine	N[C@@H](C)C(O)=O
Proline	O=C(O)[C@H]1NCCC1
Valine	N[C@@H](C(C)C)C(O)=O
Leucine	N[C@@H](CC(C)C)C(O)=O
Isoleucine	N[C@@H]([C@@H](C)CC)C(O)=O
Methionine	N[C@@H](CCSC)C(O)=O
Phenylalanine	N[C@@H](CC1=CC=CC=C1)C(O)=O
Tyrosine	N[C@@H](CC1=CC=C(O)C=C1)C(O)=O
Tryptophan	N[C@@H](CC1=CNC2=C1C=CC=C2)C(O)=O
Serine	N[C@@H](CO)C(O)=O
Threonine	N[C@@H]([C@H](O)C)C(O)=O
Cysteine	N[C@@H](CS)C(O)=O
Asparagine	N[C@@H](CC(N)=O)C(O)=O
Glutamine	N[C@@H](CCC(N)=O)C(O)=O
Lysine	N[C@@H](CCCCN)C(O)=O
Histidine	N[C@@H](CC1=CNC=N1)C(O)=O
Arginine	N[C@@H](CCCNC(N)=N)C(O)=O
Aspartic acid	N[C@@H](CC(O)=O)C(O)=O
Glutamic acid	N[C@@H](CCC(O)=O)C(O)=O
Glyceraldehyde	OC(CO)C=O
Erythrose	O=C[C@@H]([C@@H](CO)O)O
Threose	O=C[C@H]([C@@H](CO)O)O
Ribose	O=C[C@@H]([C@@H]([C@@H](CO)O)O)O
Arabinose	O=C[C@H]([C@@H]([C@@H](CO)O)O)O
Xylose	O=C[C@@H]([C@H]([C@@H](CO)O)O)O
Lyxose	O=C[C@H]([C@H]([C@@H](CO)O)O)O

Allose	O=C[C@@H]([C@@H]([C@@H]([C@@H](CO)O)O)O)O
Altrose	O=C[C@H]([C@@H]([C@@H]([C@@H](CO)O)O)O)O
Glucose	O=C[C@@H]([C@H]([C@@H]([C@@H](CO)O)O)O)O
Mannose	O=C[C@H]([C@H]([C@@H]([C@@H](CO)O)O)O)O
Gulose	O=C[C@@H]([C@@H]([C@H]([C@@H](CO)O)O)O)O
Idose	O=C[C@H]([C@@H]([C@H]([C@@H](CO)O)O)O)O
Galactose	O=C[C@@H]([C@H]([C@H]([C@@H](CO)O)O)O)O
Talose	O=C[C@H]([C@H]([C@H]([C@@H](CO)O)O)O)O
Dihydroxyacetone	O=C(CO)CO
Erythrulose	OCC([C@@H](CO)O)=O
Ribulose	OCC([C@@H]([C@@H](CO)O)O)=O
Xylulose	OCC([C@H]([C@@H](CO)O)O)=O
Psicose	OCC([C@@H]([C@@H]([C@@H](CO)O)O)O)=O
Fructose	OCC([C@H]([C@@H]([C@@H](CO)O)O)O)=O
Sorbose	OCC([C@@H]([C@H]([C@@H](CO)O)O)O)=O
Tagatose	OCC([C@H]([C@H]([C@@H](CO)O)O)O)=O
Lauric acid	CCCCCCCCCCCC(O)=O
Myristic acid	CCCCCCCCCCCCCC(O)=O
Palmitic acid	CCCCCCCCCCCCCCCC(O)=O
Stearic acid	CCCCCCCCCCCCCCCCCC(O)=O
Arachidic acid	CCCCCCCCCCCCCCCCCCCC(O)=O
Lignoceric acid	CCCCCCCCCCCCCCCCCCCCCCCC(O)=O
Cholesterol	CC(C)CCC[C@@H](C)[C@H]1CC[C@@]2([H])[C@]3([H])CC=C4C[C@@H](O)CC[C@]4(C)[C@@]3([H])CC[C@]12C

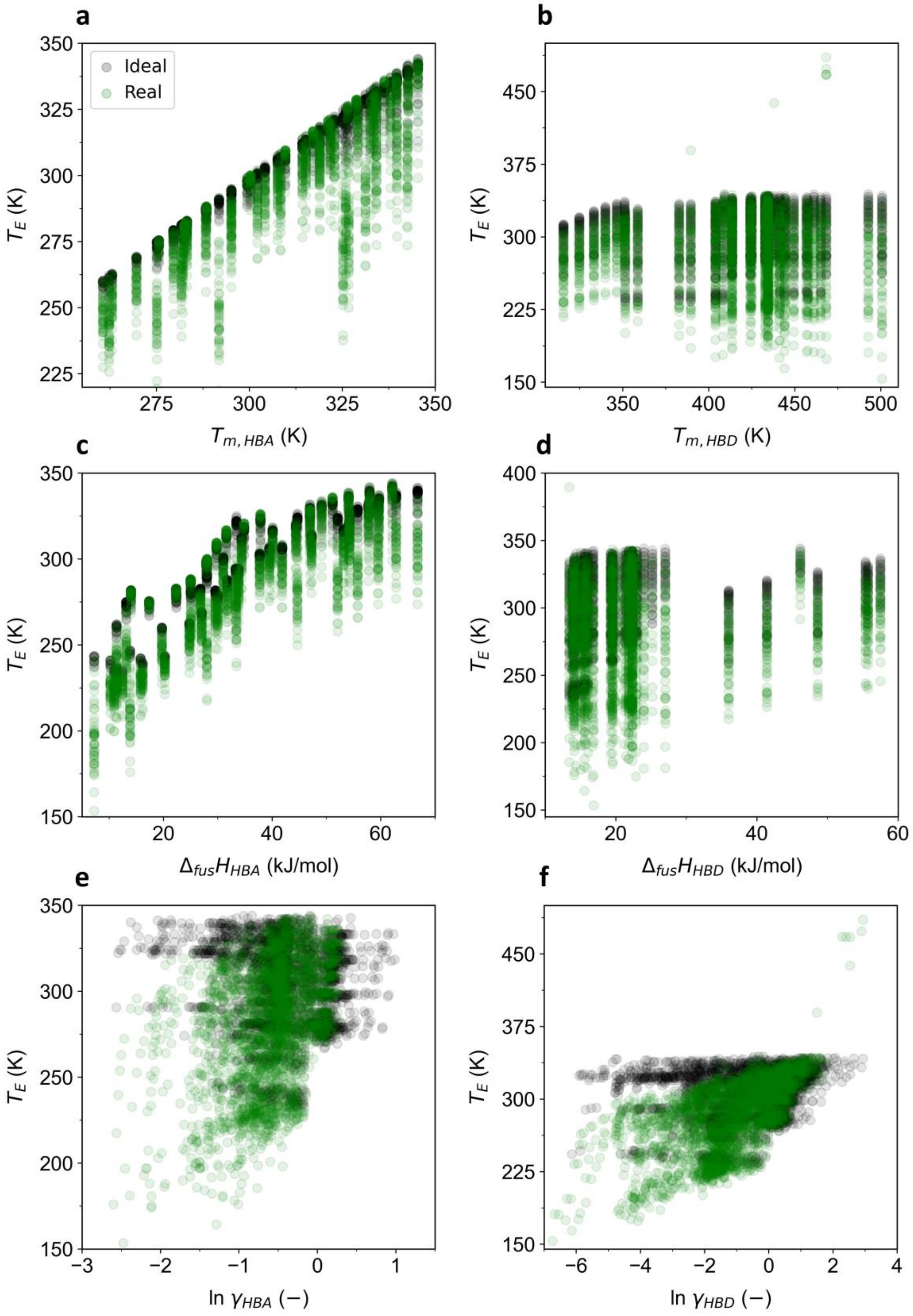

Figure S3. Correlations between (**a**) $T_{m,HBA}$, (**b**) $T_{m,HBD}$, (**c**) $\Delta_{fus}H_{HBA}$, (**d**) $\Delta_{fus}H_{HBD}$, (**e**) $\ln\gamma_{HBA}$, and (**f**) $\ln\gamma_{HBD}$ values and the T_E values of the mixtures.

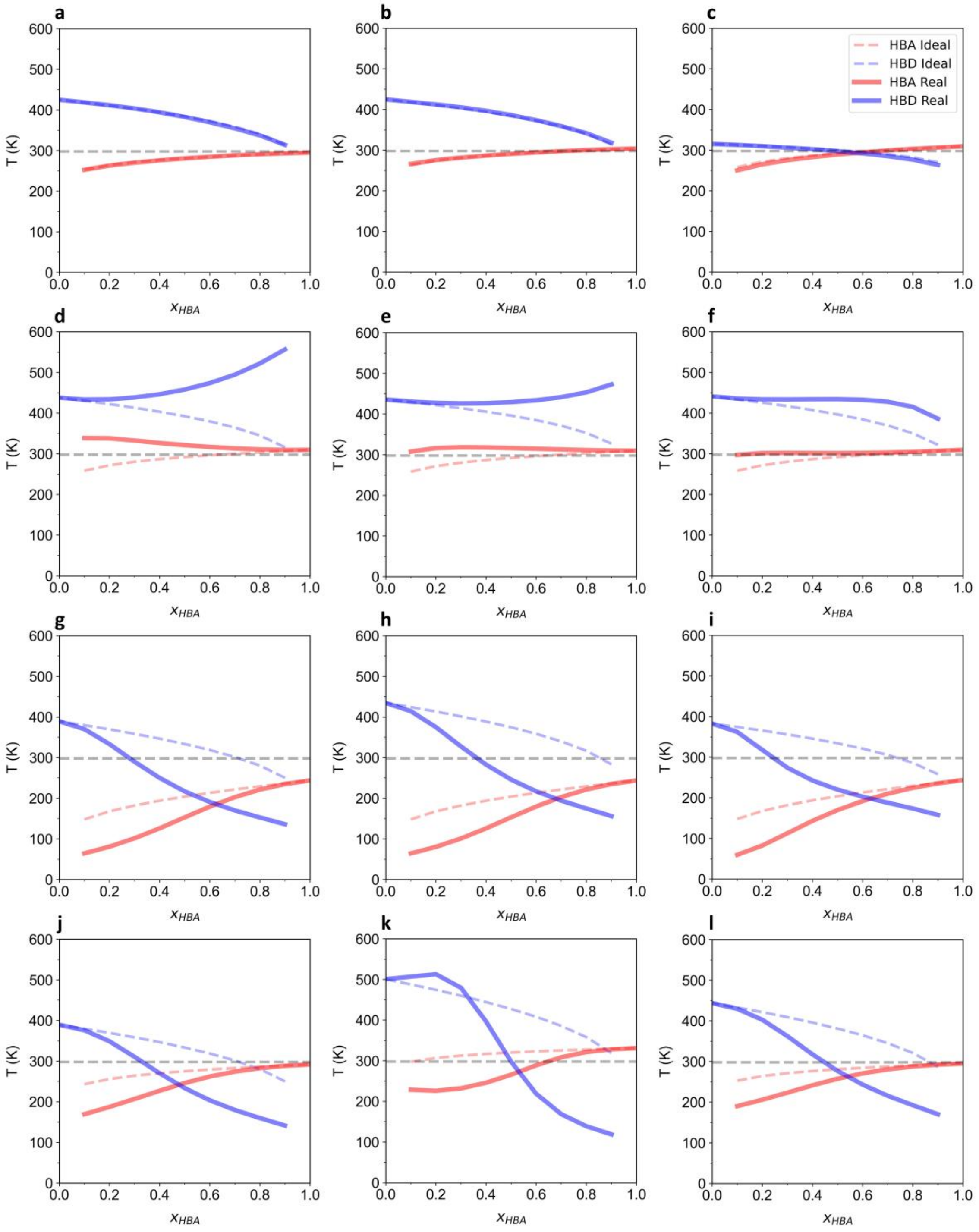

Figure S4. Additional examples of SLE phase diagrams with melting curves that (**a**–**c**) behave ideally, (**d**–**f**) exhibit positive deviations, and (**g**–**l**) show negative deviations. (**a**) Mixture 1015, (**b**) Mixture 1065, (**c**) Mixture 2993, (**d**) Mixture 2964, (**e**) Mixture 2967, (**f**) Mixture 2969, (**g**) Mixture 0, (**h**) Mixture 1, (**i**) Mixture 2, (**j**) Mixture 100, (**k**) Mixture 318, (**l**) Mixture 1012. Key details of these mixtures are given in Table S2.

Table S2. Key information on the mixtures in Figure S4.

Mixture	HBA	HBD	T_E ideal (K)	T_E real (K)
1015	1-(methylsulfinyl)undecane	lysine	294	294
1065	1-(methylsulfinyl)dodecane	lysine	303	303
2993	1-hexyl-1,3,3-trimethylthiourea	lauric acid	296	294
2964	1-hexyl-1,3,3-trimethylthiourea	glutamine	307	n.a.
2967	1-hexyl-1,3,3-trimethylthiourea	arginine	308	n.a.
2969	1-hexyl-1,3,3-trimethylthiourea	glutamic acid	308	n.a.
0	trimethylphosphine oxide	glycine	239	185
1	trimethylphosphine oxide	alanine	243	199
2	trimethylphosphine oxide	proline	240	198
100	tripropylphosphine oxide	glycine	285	242
318	triheptylphosphine oxide	aspartic acid	329	272
1012	1-(methylsulfinyl)undecane	cysteine	292	263

n.a.: not available

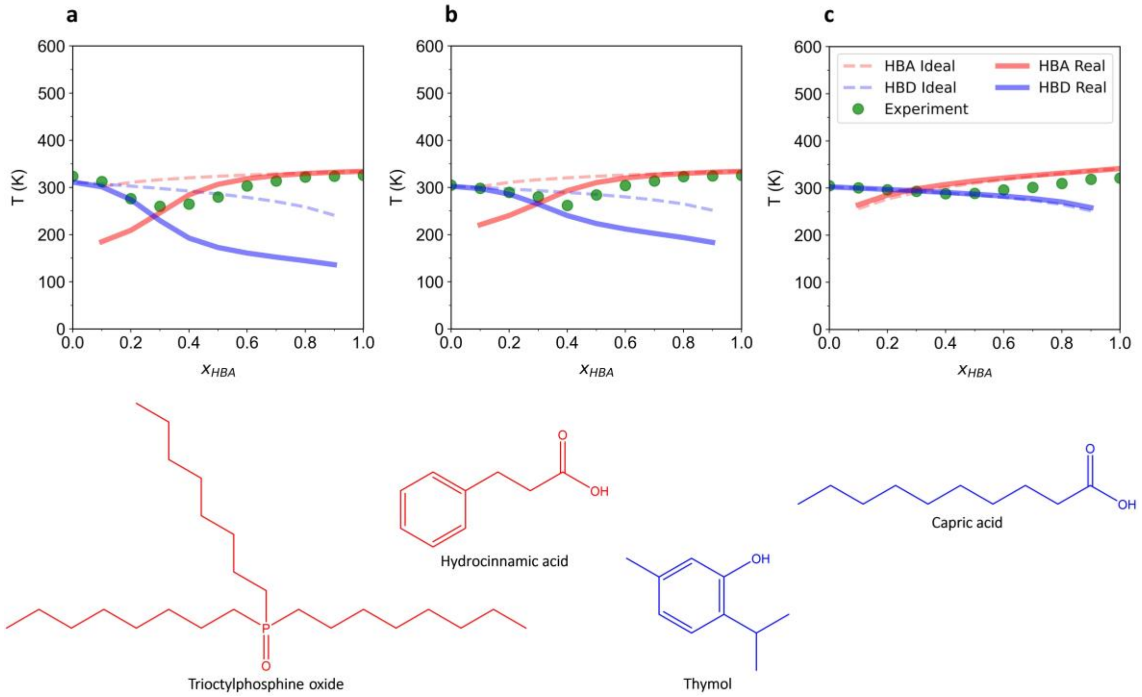

Figure S5. Estimated and experimental SLE phase diagrams for the mixture of (a) Trioctylphosphine oxide (TOPO) and Thymol, (b) TOPO and Capric acid, (c) Hydrocinnamic acid and Capric acid. Chemical structures of the compounds are shown below the diagrams. The experimental data were taken from supplementary reference [1].

Note S1

Fig. S5 shows a comparison between the estimated SLE phase diagrams for selected cases and their experimental counterparts. In the mixture of TOPO and Thymol (Fig. S5a), the melting curves from the real model exhibit a negative deviation from ideality, aligning quite well with the experimental data. For the mixture of TOPO and Capric acid (Fig. S5b), the melting curves from the real model also show a slight negative deviation from ideality and mostly coincide with the experimental data, albeit with a minor discrepancy in the resulting eutectic points. Regarding the mixture of Hydrocinnamic acid and Capric acid (Fig. S5c), the melting curves from the real model align with those of the ideal model with a few deviations from the experimental data, particularly in the HBA melting curves.

These results demonstrate the effectiveness of the model in predicting the formation of a DES. In this case, the mixture of TOPO-Thymol and TOPO-Capric acid would form DESs, while the mixture of Hydrocinnamic acid-Capric acid would likely form an ideal eutectic mixture. The estimation of eutectic points (x_E, T_E) is also quite promising with reasonable minor deviations from the experimental data. The estimated values are practically useful for navigating the choice of DESs out of the vast chemical space and guiding the determination of SLE phase diagrams for thermally unstable compounds. Future enhancements in prediction accuracy can be achieved by refining the model and expanding the training data.

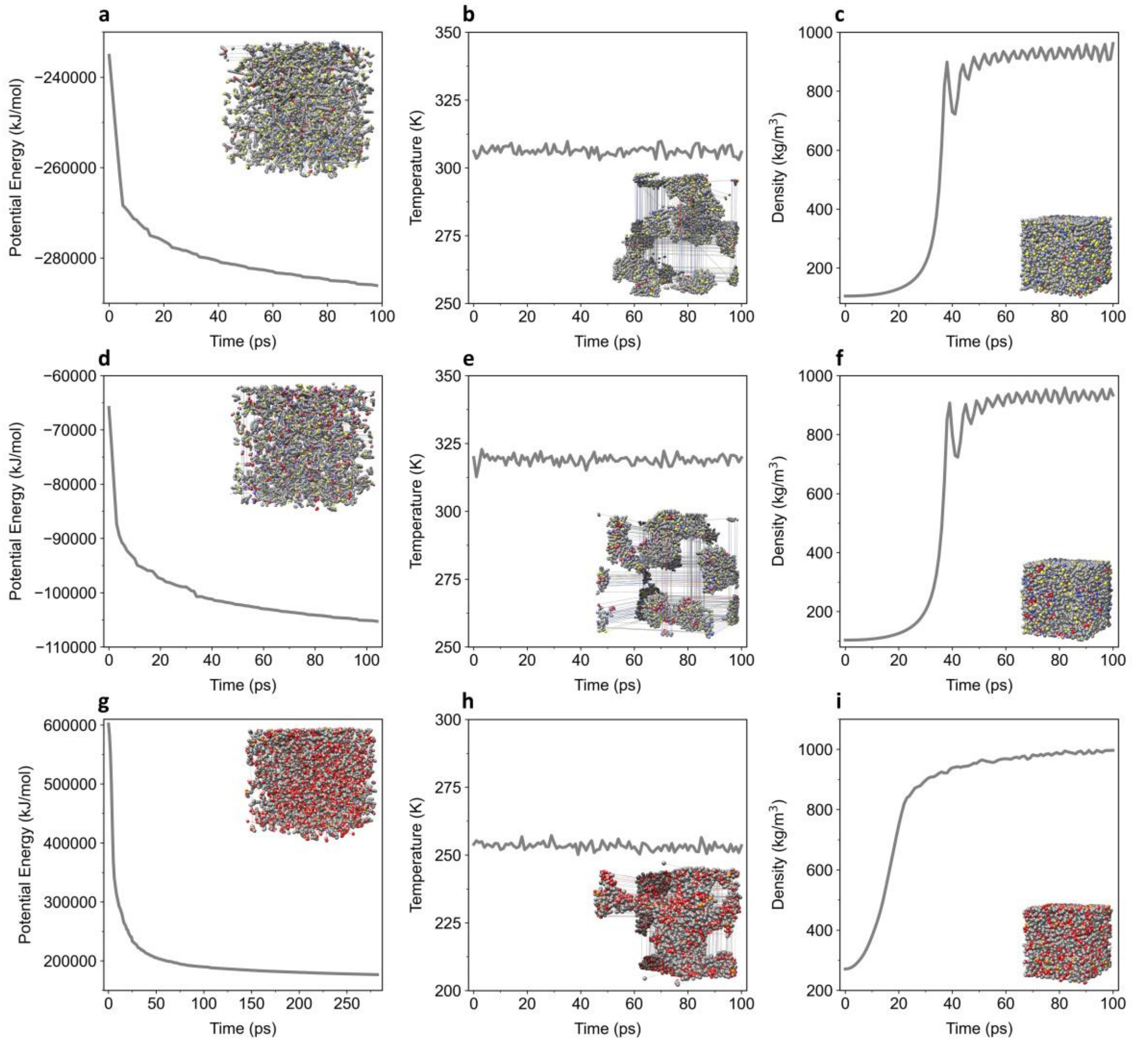

Figure S6. Setup of the simulation systems prior to the MD productions. (**a**) energy minimization, (**b**) NVT equilibration, and (**c**) NPT equilibration of Mixture 2996. (**d**) energy minimization, (**e**) NVT equilibration, and (**f**) NPT equilibration of Mixture 2650. (**g**) energy minimization, (**h**) NVT equilibration, and (**i**) NPT equilibration of Mixture 171. The inset depicts molecular visualizations at the end of each simulation step.

Supplementary References

1. Schaeffer, N. et al. Non-ionic hydrophobic eutectics – versatile solvents for tailored metal separation and valorisation. *Green Chemistry* 22, 2810–2820 (2020).
